# Probabilistic outlier identification for RNA sequencing generalized linear models

**DOI:** 10.1093/nargab/lqab005

**Published:** 2021-03-01

**Authors:** Stefano Mangiola, Evan A Thomas, Martin Modrák, Aki Vehtari, Anthony T Papenfuss

**Affiliations:** The Walter and Eliza Hall Institute, Parkville, Victoria, 3052, Australia; Department of Medical Biology, University of Melbourne, Melbourne, Victoria, 3010, Australia; The Walter and Eliza Hall Institute, Parkville, Victoria, 3052, Australia; The Florey Institute of Neuroscience and Mental Health, The University of Melbourne, Victoria, 3010, Australia; Institute of Microbiology of the Czech Academy of Sciences, Prague, 1083, Czech Republic; Department of Computer Science, Aalto University, Aalto, FI-00076, Finland; The Walter and Eliza Hall Institute, Parkville, Victoria, 3052, Australia; Department of Medical Biology, University of Melbourne, Melbourne, Victoria, 3010, Australia; Peter MacCallum Cancer Centre, Melbourne, Victoria, 3000, Australia; Sir Peter MacCallum Department of Oncology, University of Melbourne, Melbourne, Victoria, 3010, Australia; School of Mathematics and Statistics, University of Melbourne, Melbourne, Victoria, 3010, Australia

## Abstract

Relative transcript abundance has proven to be a valuable tool for understanding the function of genes in biological systems. For the differential analysis of transcript abundance using RNA sequencing data, the negative binomial model is by far the most frequently adopted. However, common methods that are based on a negative binomial model are not robust to extreme outliers, which we found to be abundant in public datasets. So far, no rigorous and probabilistic methods for detection of outliers have been developed for RNA sequencing data, leaving the identification mostly to visual inspection. Recent advances in Bayesian computation allow large-scale comparison of observed data against its theoretical distribution given in a statistical model. Here we propose ppcseq, a key quality-control tool for identifying transcripts that include outlier data points in differential expression analysis, which do not follow a negative binomial distribution. Applying ppcseq to analyse several publicly available datasets using popular tools, we show that from 3 to 10 percent of differentially abundant transcripts across algorithms and datasets had statistics inflated by the presence of outliers.

## INTRODUCTION

The analysis of the relative gene transcriptional abundance through RNA sequencing has been valuable for molecularly characterizing biological systems. The sequencing of RNA involves sampling from the population of transcripts present in solution at the time of RNA extraction; the number of sequenced RNA molecules reflects the relative proportion/concentration of each transcript. A large number of methods for differential transcript-abundance at the gene level (i.e. differential expression) analysis have been designed and adapted for RNA sequencing data (1). A popular modelling choice for RNA sequencing data is the negative binomial framework. The negative binomial distribution has independent parameters for mean and overdispersion and can be thought as an extension of the Poisson distribution, where the mean parameter is generated from a gamma distribution. The negative binomial distribution can be interpreted as a model of two types of variability: (i) the biological variability in mRNA synthesis/degradation rates between replicates (the gamma distribution) and (ii) the intrinsic variability in mRNA counts given constant synthesis/degradation rate and the inherently imperfect efficiency of mRNA extraction and sequencing (the Poisson distribution).

The most popular algorithms for differential gene transcriptional abundance analysis based on negative binomial data assumptions rely on generalized linear models. To regularize estimates of mean and variance, the quadratic association between the two is often modelled (2–5). For example, edgeR (2) estimates common and feature-wise dispersion through empirical Bayes and shrinks the dispersions for each gene toward a common prior using weighted conditional log-likelihood. Similarly, DeSeq2 (3) moderates feature-wise dispersion estimates toward a common trend by a geometric normalization strategy. Although frequentist methods have been historically the most popular, Bayesian statistics have been also widely employed for transcriptomics analyses (6) based on negative binomial frameworks. Zhao *et al.* proposed an integrated model for gene transcriptional abundance quantification and differential analyses based on a negative binomial framework (7). The notion that joint modelling outperforms independent maximum likelihood estimation is further supported in the literature (8,9). An extensive discussion on parametric and non-parametric prior choice for Bayesian framework of RNA sequencing count is provided by Van De Wiel *et al.* (10).

Although most gene counts are well-fitted by the negative binomial distribution, the underlying gamma distribution has thin tails and thus is not robust against the presence of unmodelled large-scale biological variability. Larger than expected variability results in some biological replicates (outliers) having disproportionate influence on the final inference, increasing both false positives and false negatives. However, adverse consequences go beyond differential abundance classification (e.g. false discovery rate < 0.05). When focusing on specific transcripts, inflated fold changes and deflated *P*-values communicate a false perception of certainty about the association between transcript abundance and the factor of interest; even if the outliers-free data provide a false discovery rate lower than the user defined threshold. When performing summary analyses (e.g. gene enrichment), inflated statistics can affect methods based on gene rank and/or on fold changes. The attention that several popular methods (3,11,12) give to outlier detection provides evidence for the importance of the matter. Examples exist of methods that use robust versions of the negative binomial framework (12–14). More broadly, a large number of robust (long-tailed) gamma-compound distributions exist (15–17); however, the implementation of statistical models from many of those is not trivial and often require non-efficient computations as a closed-form of the probability density does not always exist. Considering that by far the most used methods for differential gene transcriptional abundance are edgeR (2) and DESeq2 (3) (23rd and 26th top downloaded packages in R/Bioconductor repository; bioconductor.org/packages/stats accessed June 2020), to develop an independent evaluation tool for identifying transcripts that may have unreliable statistics is extremely relevant.

Although the analysis of errors between the inferred theoretical distribution and the data (i.e. residuals) is possible, this is not suitable for heteroscedastic data such as RNA sequencing, and it relies on a sufficiently large biological replication and would require care to consider the information about overall uncertainty of the inferred model. For example, DESeq2 uses the Cook’s distance (18,19) to identify potential outlier data points. However, this implementation does not control for false positives for multiple inference, relies on a minimum biological replication and can be applied only to linear models with discrete covariates (3). A rigorous, probabilistic and automated quality-control tool for detecting data points (i.e. biological replicate/transcript pairs) that do not follow a negative binomial regression model is currently missing. Bayesian inference provides a robust methodology to simulate the theoretical data distribution according to the joint inferred model, which includes the integrated uncertainty of the hierarchical parameters (i.e. a posterior predictive check), and therefore is suitable for low-data regimes. The observed data can be mapped against the theoretical data distribution and posterior quantiles of the observed data points can be computed. If those quantiles are close to extremes (0 or 1), it indicates there is a possible mismatch between the model and the data. Furthermore, with the Bayesian inference framework it is possible to re-fit the model omitting the suspected outlier data-points, avoiding a biased inference. Recent computational advances on the sampling of multidimensional posterior distributions (dynamic Hamiltonian Monte Carlo (20) and variational Bayes (21,22)) allow the efficient joint hierarchical modelling of large scale RNA sequencing datasets. Here we describe ppcseq, a quality-control tool based on the probability framework Stan (23) that is able to (i) model RNA sequencing gene transcriptional abundance using hierarchical negative binomial regression; (ii) produce theoretical data distribution with and without possible outliers; and (iii) flag data points that fall outside the credible interval (for an arbitrary quantile, dictated by the false positive rate) of their theoretical distributions. This information helps the user flagging transcripts that need further attention and/or reanalysis. Applying ppcseq to selected publicly available datasets, we identified up to 10% of transcripts with fold-change inflated by the presence of outliers.

## MATERIALS AND METHODS

### Iterative outlier detection

**Figure 1.**
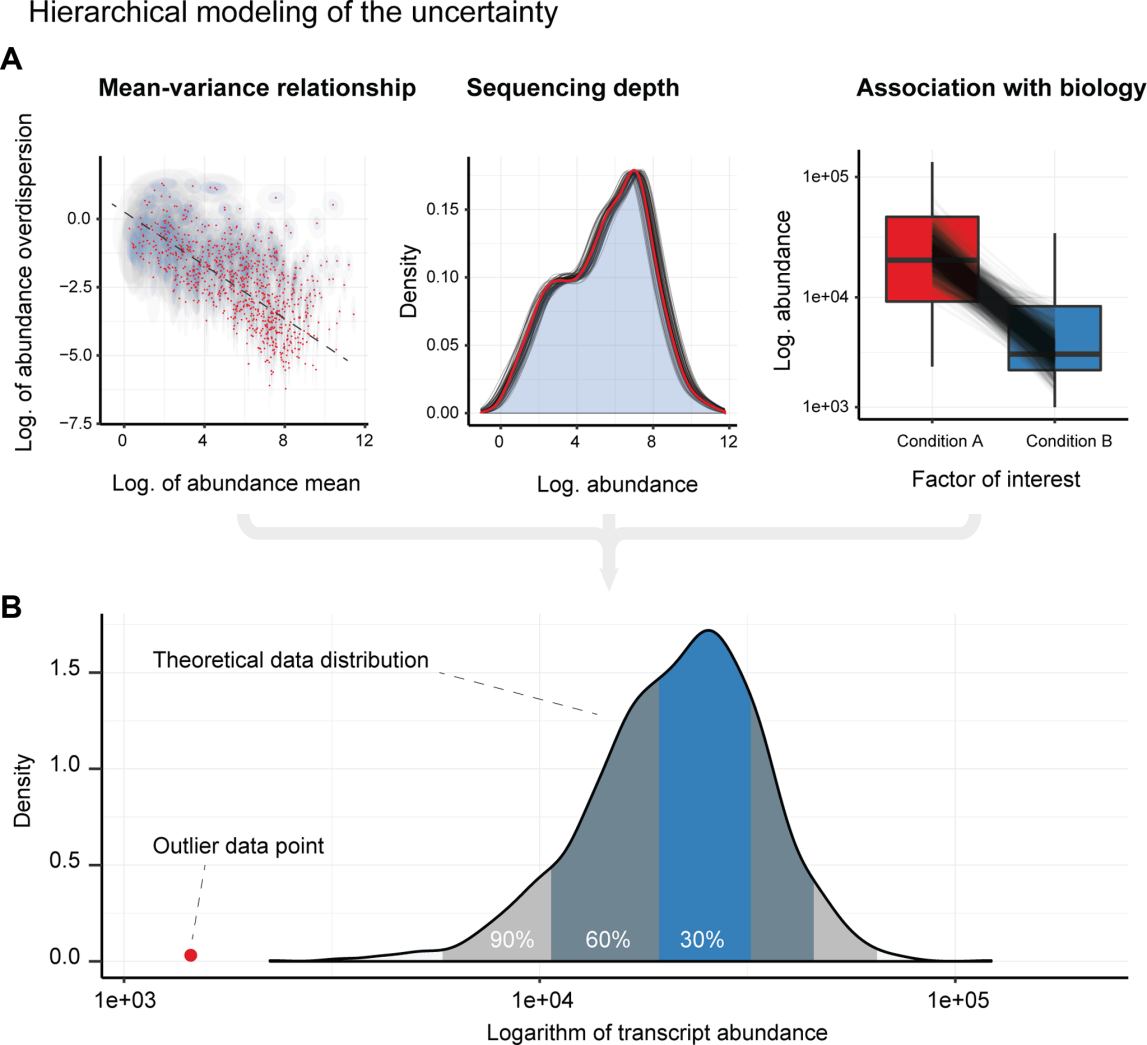
Graphical example of the estimation of the theoretical data distribution. The theoretical data distribution is estimated for each biological-replicate/transcript pair from the joint posterior distributions. (**A**) Left: the uncertainty of transcript-wise abundance baseline, where log-overdispersions are modelled in association with their log-mean. The point estimates are coloured in red, while the ellipses represent the 2D credible intervals 40% (blue) and 95% (grey); Middle: the density histogram of the posterior expected values for all genes for one biological replicate, adjusted by the exposure parameter (95% credible interval). The overlapping densities represent the uncertainty of the sequencing depth for one biological-replicate, modelled by the exposure parameter. The red curve corresponds with the adjustment for the mean of the posterior probability of the inferred exposure rate; Right: the uncertainty of the expected abundance values (95% credible interval) of a single transcript across two experimental conditions. The boxplots visualize the observed data distribution, while the black lines visualize the posterior densities of expected abundance according to the linear model. (**B**) An illustrative example of the theoretical transcript abundance distribution that is estimated for a biological replicate/transcript pair. Shaded regions correspond to central credible intervals of the distribution.

**Figure 2.**
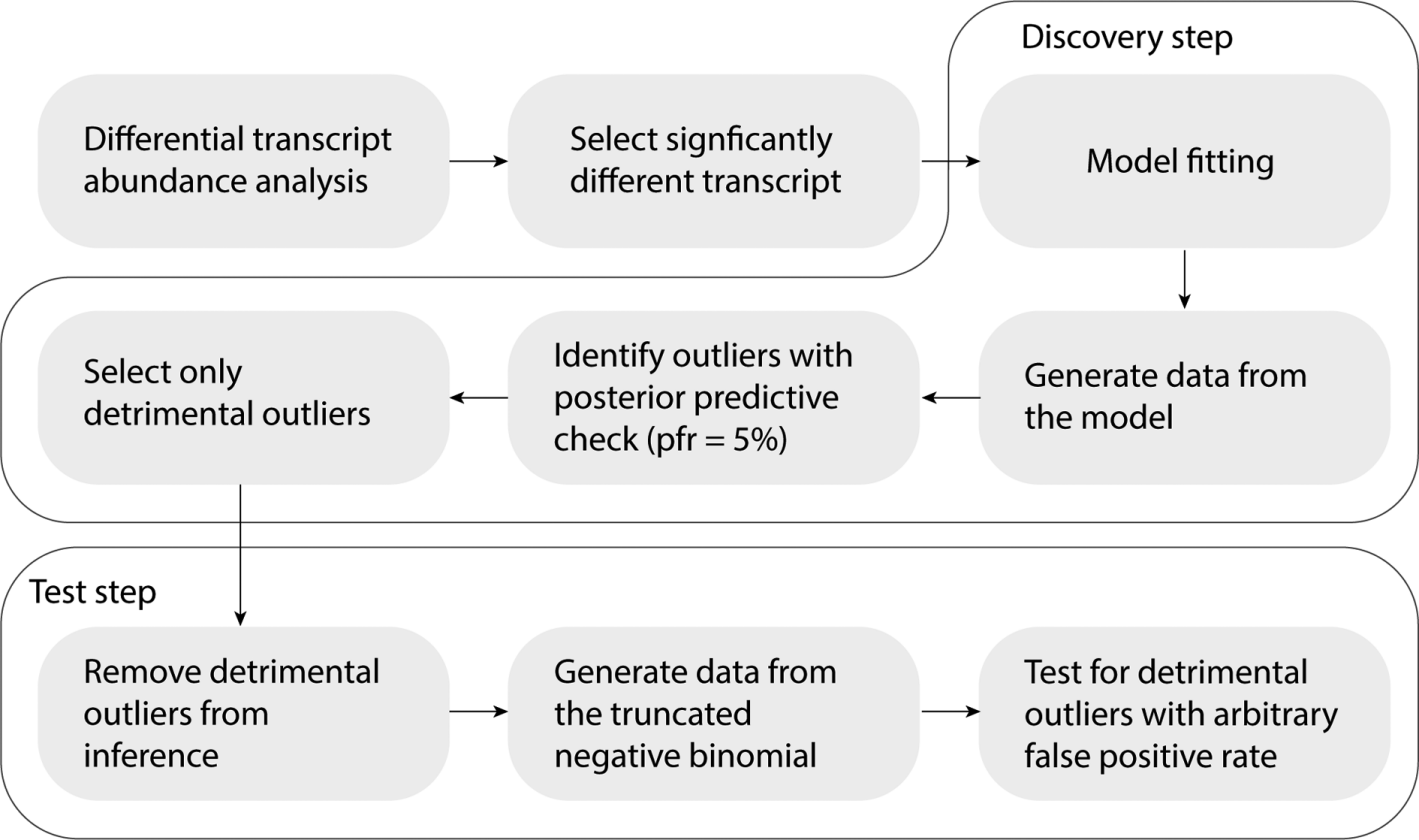
Flow chart of the two-step strategy for outlier detection, including discovery and test steps. Because a model that includes outliers is ill-posed by definition, a first discovery step allows the flagging of potential outliers with relaxed criteria, while a second test step allows the evaluation of those potential outliers against a model fitted without them. The workflow includes a preliminary independent estimation of differential gene transcriptional abundance with methods such as edgeR (2) or DESeq2 (3). Genes which outliers will be selected from the significance rank. The first step of the outlier identification includes the fitting of the user-defined linear model on the user gene-selection. Then, the theoretical data distribution is generated from the join posterior, and genes are flagged as potential outliers with a default false positive rate threshold of 5%. Of those, only detrimental outliers (see ‘Materials and Methods’ section) are flagged. The test step includes the removal of possible detrimental outliers from the data, and the fit of the same model, compensating for data truncation. Then, the theoretical data distribution is generated from the join posterior and potential outliers are checked against, with a better calibrated false positive rate (0.01 by default).

To identify the transcripts that partially violate the negative binomial assumption, three types of uncertainty are modelled jointly from the data (Figure 1): (i) the mean abundance and overdispersion of transcripts, and their log-scale-linear association; (ii) the effect of sequencing depth; and (iii) the association between transcript abundance and the factors of interest. The inference workflow consists of two iterative steps (Figure 2): first a ‘discovery’ step identifies potential outliers, and second, the probability of a model excluding those data points is estimated in a ‘test’ step. The motivation is 2-fold. First, after some outliers have been identified, the model needs to be refitted as those outliers might have skewed the initial estimates noticeably. In theory, this process would need to be iterated until convergence; however, in our analyses across six representative datasets from public sources we found that two iterations were always enough, as no transcript was identified including outliers that were not identified in the first discovery phase. Second, the stringency of the check for outliers can be set separately for each step. That is, we can identify potential outliers with a loose criterion (by default 5% false positive rate across all biological-replicate/transcript pairs), refit the model and then check whether those outliers are classified as such against the refitted model but with more stringent criteria (by default 1% false positive rate, internally adjusted by the number of biological replicates, so to control the false positive rate of a transcript including an outlier data point), letting us improve both sensitivity and specificity of the method.

In the first ‘discovery’ step, the model is fitted to a list of previously identified differentially abundant transcripts at the gene level. The transcript abundance information for a set of genes whose abundance is highly conserved (i.e. housekeeping) is also used, for inferring the effect of sequencing depth for each biological-replicate (Supplementary Methods). New data are generated from the fitted model, providing the theoretical range of values for each data point. All observed read counts that are outside the 95% posterior credible interval are quarantined as possible outliers. In the second ‘test’ step, the model is fitted again excluding the deleterious outlier data points that would inflate the estimated difference between conditions (i.e. only the combinations (i) higher than the upper quantile of the credible interval when the transcript abundance is estimated to have increased; or (ii) smaller than the lower quantile when the transcript abundance is estimated to have decreased) using a truncated negative binomial distribution at 2.5% and 97.5% quantiles (Supplementary Figure S1). New theoretical data distributions are generated from the second fitted model, and all the observed read counts (including possible deleterious outliers quarantined from the inference) are tested against these, using a credible interval that matches the user-selected false positive rate, assuming the remaining data are generated by a pure negative binomial process. Given the desired false positive rate (1% by default), the interval width is taken as $\frac{{fpr}}{{2\ {n_{outliers}}}}$ where the factor of two compensates for unidirectionality of the tests (just for deleterious outliers). A Bayesian inference probabilistic network is used to model the raw read counts, based on a negative binomial regression (Supplementary Methods - Equations 1–7; Supplementary Figure S2).

### Posterior probability distribution sampling and approximation strategies

To infer and sample from the joint posterior distribution of all parameters, the Bayesian probabilistic framework Stan was used (23). With our algorithm, it is possible to explore the posterior distribution both with dynamic Hamiltonian Markov-chain Monte Carlo sampling or with variational Bayes (approximating the posterior distribution with multivariate normal) (24).

When the number of draws from the posterior distribution needed to calculate the credible internals of the theoretical data distribution is too large from a practical standpoint, this can be approximated with a semi-analytical method (referred here to approximated credible interval). The credible interval of the theoretical distribution of each observed data point can be estimated using the optim R utility (25) to find the mean of the *N* theoretical credible intervals (both upper and lower; accordingly with the user-selected false positive rate) given the mean, exposure and overdispersion parameter *N* draws.

### Calibration and accuracy test

To test the accuracy of the outlier inference, we produced simulated data from the joint posterior distribution fitted on real data (26), including 339 transcripts to be tested (result of edgeR analysis; FPR < 0.05) across 21 biological replicates. Briefly, we performed differential transcript-abundance analysis at the gene level of this dataset using edgeR (2) and identified potential differentially abundance transcripts (FDR < 0.05) according to a linear model including risk as the only covariate. Those transcripts were modelled with our Bayesian inference model, and the posterior distribution was used to generate simulated data that come from a pure negative binomial generative process and have all the biological and experimental properties of the source experimental dataset. For a random selection of 50% of those transcripts, we injected one outlier for one randomly selected biological replicate, characterized by a right-quantile distance 1–10^−10^ of the theoretical distribution of the selected data points.

We then used these simulated datasets to calculate the false positive and false negative rate testing for 18 user-selected false positive rate thresholds, ranging from 0.2% to 10%, replicating each run three times for a total of 54 runs. We then calculated (i) the proportion of transcripts labelled as containing outliers and compared them with the nominal false positive rate threshold and (ii) the false negative calls per each nominal false positive rate threshold.

## RESULTS AND DISCUSSION

### Model calibration

**Figure 3.**
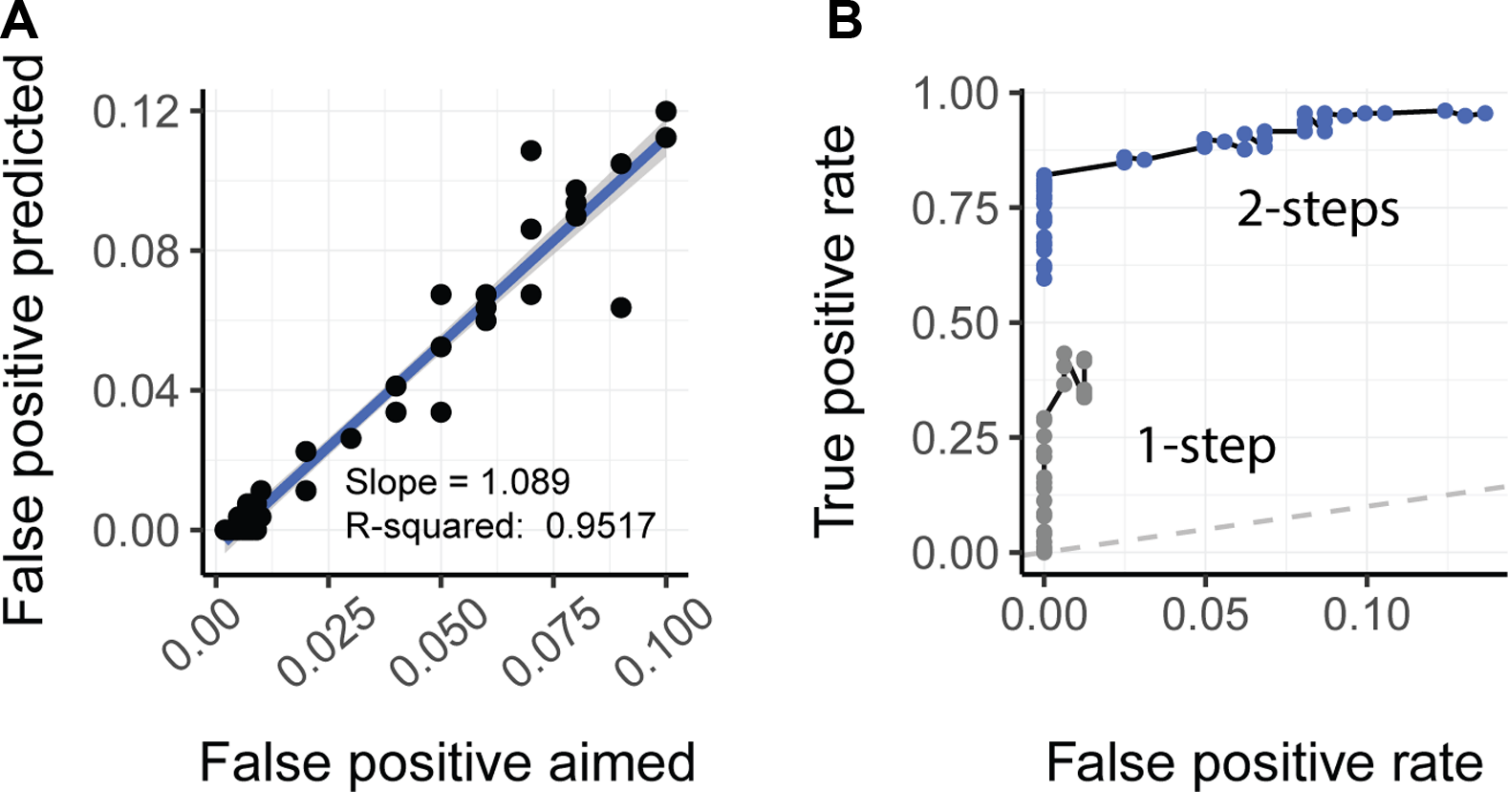
Calibration and performances of the ppcseq algorithm. (**A**) Scatter plot showing good calibration of false positive rate, representing the linear association between the user defined false positive rate and the false positive rate that the model identified on a simulated dataset with no outliers. The statistics are relative to a linear interpolation of the data using the lm function in *R*. (**B**) Receiver operating characteristic (ROC) showing the performance of classification of transcript including outliers. The data points (blue) include only inference with the false positive aimed within a meaningful range for standard applications (from 0.002 to 0.1). For proving that the two-step-strategy (discovery and test) is highly beneficial for accurate outlier classification, for each two-step-strategy classification (blue points) the one-step-strategy counterpart is shown (grey points; obtained using theoretical data distribution from the first discovery step). The one-step-strategy (discovery only) shows lack in sensitivity, due to inflated variance of the inferred theoretical data distributions, driven by the presence of outliers.

Testing on simulated data showed that the model is well-calibrated for false positive rate (Figure 3A). The correlation across runs with a wide range of false positive rate thresholds (from 0.001 to 0.1) is close to 1 with a *R*-square of 0.95. The false negative rate for outliers outside the credible interval is 0.37 for an aimed false positive rate of 5%, tested against 339 genes across 21 biological replicates (for a total of 7119 inferences; Figure 3B, blue points). The false positive rate is well calibrated also for the use of multiple-covariate linear models (Supplementary Figure S3).

Although our model is well-calibrated against data generated from a negative binomial process, care is needed into making claims about probabilities. In the first discovery step, we quarantine data based on the 95th percentile, although this interval is an estimate, given that the presence of outliers makes the numerical generative process not negative binomial by definition. In the second test step, the modelling of the data without quarantined points allows a much better estimation of the a-posteriori probabilities and the false positive rate. For the estimation of a truncated negative binomial, we observed that a non-truncated negative binomial distribution under-estimates the overdispersion for data truncated at the 95th percentile to an approximately constant degree. The overdispersion parameter σ (with over-dispersion being ${e^\sigma }$) has a 74% reduction across all mean/sigma combinations that are typical of RNA sequencing data (Supplementary Figure S1).

The centrality of the use of an iterative strategy including a truncated distribution is supported by attempts to identify outlier data points with only one passage (i.e. discovery stage; Figure 3B, grey points) with an approximate false positive rate of 0.2 to 10%. Using this false positive rate, almost no outliers could be detected, mainly because the presence of deleterious outliers significantly inflates the change in gene transcriptional abundance between the two conditions, biasing the inference.

### Application to real data, user interface and generated graphics

**Figure 4.**
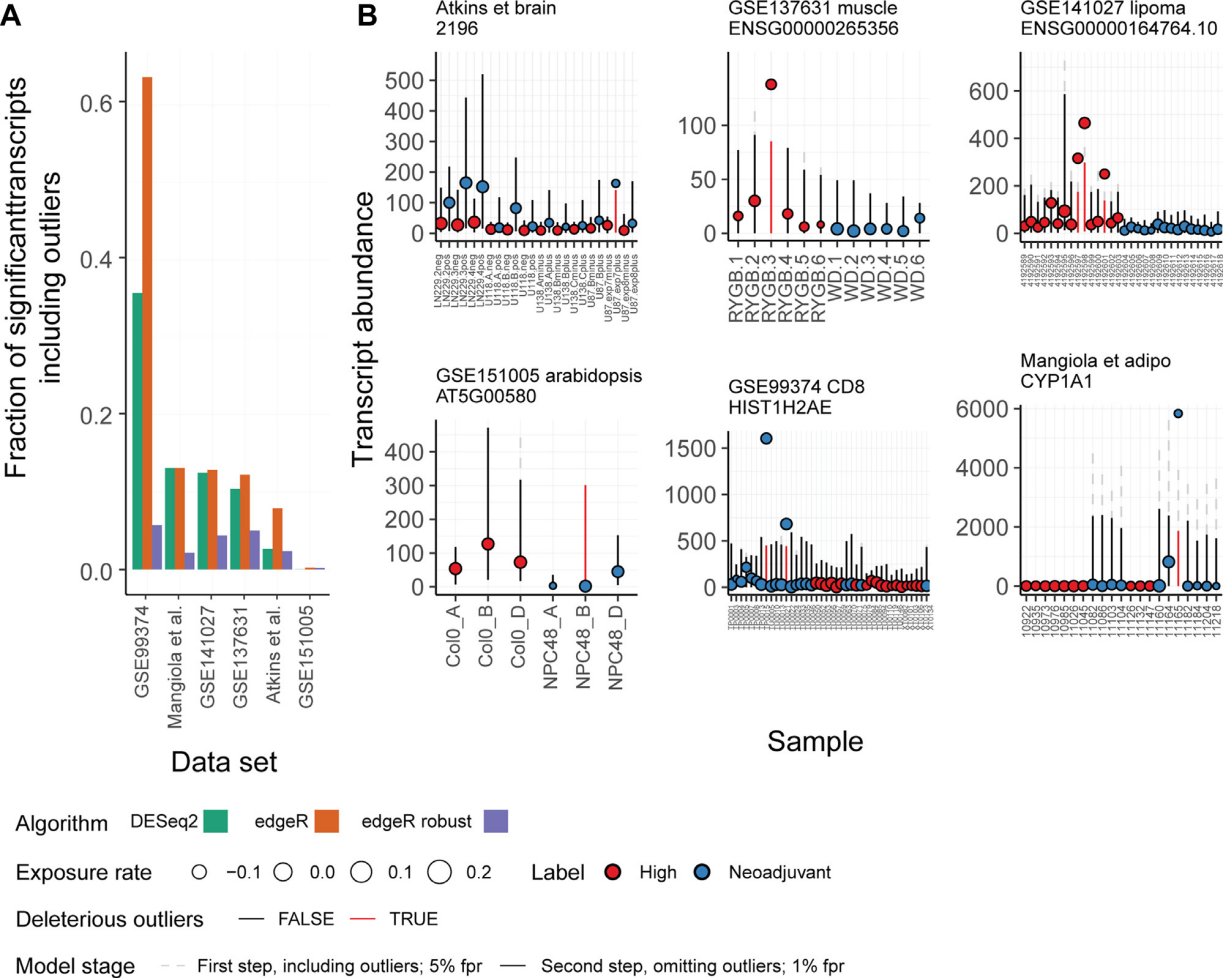
Outlier detection on real-word data. (**A**) Bar plot representing the fraction of differentially transcribed genes (inferred by edgeR and DESeq2 according to recommendations (32,33)) that include outliers for six datasets collected from public repositories. (**B**) Visualization produced by ppcseq R package of the top differentially transcribed genes for each dataset, which included outliers. The colour coding represents the treatment regime, the error bars represent the credible interval of the theoretical data distribution, the size of the points is proportional to the inferred sequencing depth factor (exposure rate). The dashed error bars represent the 95% credible interval of the theoretical data distributions including outliers (first discovery stage), while the solid error bars represent the 99% credible interval (user defined parameter) data distribution excluding outliers, derived from truncated (at 95ft percentile) negative binomial distributions. The red error bars represent the outlier observations that do not fit the model.

The application of our model to a series of datasets gathered from public sources, including GSE137631 (27), GSE141027 (28), GSE99374 (29), GSE151005 (30), Mangiola_2018 (26) and Atkins_2019 (31), revealed that a median of 10.6%, 3.3% and 10.4% of differentially abundant transcripts inferred by edgeR, the robust edgeR implementation (using estimateGLMRobustDisp) and DESeq2 respectively (using recommended analysis pipelines for data filtering, normalisation and modelling (32,33)) had inflated statistics caused by the presence of outliers (Figure 4A). The analyses were performed using tidybulk framework (34) and broom (35). The algorithm DESeq2 did not detect outliers-including genes among the significant calls for most test datasets, except for three genes for the Mangiola_2018 dataset (26). Both for edgeR and DESeq2, five of the six data sets had the top ranked transcript that included one or more deleterious outliers placed within the top-100 differentially abundant transcripts, and for three of the datasets within the top-10 (Supplementary Table S1). On average, the decrease of log fold change of the transcripts including outliers ranged from 1.42 to 4.51 times across the six data sets for edgeR analyses and 1.57 to 2.44 for DESeq2. The robust implementation of edgeR ranked outlier-including genes among the top-100 for four datasets, and among the top-10 for one dataset (Supplementary Table S1). On average, the decrease of log fold change of the transcripts including outliers ranged from 1.06 to 1.76 times across the six data sets for the robust edgeR implementation.

The R package ppcseq provides a summary table that includes outliers detected for each transcript and a summary annotated plot underlying the theoretical data distribution with the raw data (Figure 4B). Within the scatter plot, point size represent the relative sequencing depth, solid error bars represent the data credible interval according to the user-selected false positive rate (number of false positive calls divided by all positive calls), while dashed line represent credible intervals of the outliers-including model (first step). The API allows to input gene transcriptional abundance data, sample annotations and estimates from a previous analysis (e.g. with edgeR or DESeq2) in a tidy format. The input to the identify_outliers is a data frame as shown in Table 1. The input will be read by the function identify_outliers that also takes a formula, expressing the same design used for the analyses up to that point. As example, we provide the code to execute ppcseq from a tidy data frame of counts (Table 2) and edgeR or Deseq2 R objects (code snippets 1 and 2, respectively).

**Table 1.** Example of input dataset for the function ppcseq::identify_outliers

Sample	Transcript	count	Factor or interest	*P*-value	Do check
<CHR or FCTR>	<CHR or FCTR>	<INT>	<CHR or FCTR>	<DBL>	<BOOL>

**Table 2.** Example of count dataset for the join with edgeR or DESeq2 R objects

Sample	Transcript	count	Factor or interest
<CHR or FCTR>	<CHR or FCTR>	<INT>	<CHR or FCTR>

Code snippet 1

edgeR_fit %>%

# Format

as.data.frame %>%

as_tibble(rownames = ‘ens_iso’) %>%

mutate(significant = FDR<0.05) %>%

# Join with counts

left_join(counts) %>%

# Run ppcseq

identify_outliers(∼ type,sample,ens_iso,count,.significance = PValue,.do_check = significant)

Code snippet 2

deseq2_fit %>%

# Format

tidy() %>%

filter(p.adjusted %>% is.na %>% '!' & term = = ‘type_Lipoma_vs_LipoControl’) %>%

mutate(significant = p.adjusted<0.05) %>%

# Join with counts

left_join(counts, by = c (‘gene’ = ‘ens_iso’)) %>%

# Run ppcseq

identify_outliers(∼ type,sample,gene,count,.significance = p.value,.do_check = significant)

### Variational Bayes and approximation of the credible interval do not compromise the inference

The test runs performed with increasing level of parallelization (from 2 to 16 physical cores) show a gradual speed up to three times for the Hamiltonian Monte Carlo sampling (Supplementary Figure S4). Compared to the Hamiltonian Monte Carlo sampling, variational Bayes showed speedup from 2- to 6-folds depending on the level of parallelization (of the alternative Hamiltonian Monte Carlo sampler, from 16 to 2 physical cores respectively; Supplementary Figure S4).

**Figure 5.**
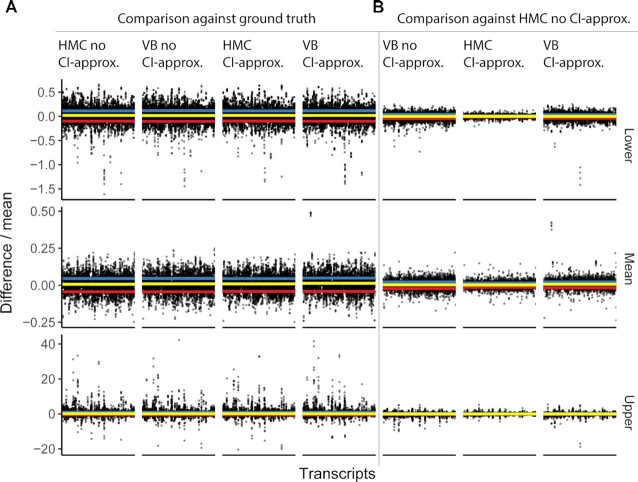
Evidence that the use of both variational Bayes and credible interval (CI) approximation does not significantly affect the inference compared with the Hamiltonian Monte Carlo sampler. These tests were performed on simulated data from the joint posterior distribution fitted on real data (26) (see ‘Materials and Methods’ section). Each data point represents the inference for one gene. The *y* axis represents the scaled (by mean) difference of each method combination (specified in the header of the vertical facet strips) and the ground truth (specified in the panel headers). The three horizontal facet strips (lower, mean, and upper) include the inferred mean, and lower and upper quantiles (95% credible interval) of the theoretical data distributions. The yellow horizontal line corresponds to the median error, the blue and red lines correspond to the upper and lower standard deviation. (**A**) Comparison of the ground truth (simulated data) with (from left to right) Hamiltonian Monte Carlo sampling, variational Bayes, Hamiltonian Monte Carlo with credible interval approximation, and variational Bayes with credible interval approximation. (**B**) Comparison of the Hamiltonian Monte Carlo sampling (without any approximation) with variational Bayes (with and without credible interval approximation) and Hamiltonian Monte Carlo sampling with credible interval approximation.

The approximation of credible intervals of the theoretical data distribution (see ‘Materials and Methods’ section) is consistent with the estimation through posterior sampling (Figure 5A and Supplementary Figure S5), with a relative error of the distribution mean (average across all approximation combinations) of 0.10, a relative error of the lower quantile of 0.04 and of the upper quantile of 0.71. The use of both variational Bayes and credible interval approximation do not affect the inference compared with the Hamiltonian Monte Carlo sampler (Figure 5B), and bias in the under-estimation of the negative binomial variance is not noticeable. Overall, this efficient approach lets us restore almost exactly the posterior intervals (for HMC using 300 warm up iterations and convergence diagnostics Rhat of 1 for most parameters with a maximum of 1.01; for variational Bayes, with max 50000 iterations, and with stopping tolerance 0.01). Although the Pareto *k* diagnostic value (36) is ∼7 for the dataset tested, indicating that the variational Bayes approximation is not close to the true posterior, there is no practical difference for quantities of interest when compared to results from dynamic Hamiltonian Monte Carlo. Variational Bayes is the default approach for both discovery and test steps, but Markov chain Monte Carlo is also available.

## CONCLUSIONS

Differential transcript-abundance analyses at the gene level are key in many areas of biology, and often studies include a limited number of biological replicates. In these cases, the effect of outlier observations can have a disproportionate impact on the prioritization of differentially abundant transcripts. This is important both when specific transcripts are of interest because it leads to inflated belief of certainty about biological associations, and when global characteristics of the data are of interest as it affects analyses relying on gene ranks and fold changes, such as gene enrichment. Methods such as the robust implementation of edgeR, which we strongly recommend, can decrease the impact of outlier data points on the statistical inference of three folds on average. However, our analyses show that the issue is not fully eliminated; furthermore, the user remains unaware of which gene-transcripts include outliers and therefore is unable to judge the impact of outliers on the method of choice for any specific dataset. Also, when specific genes are of interest for a follow-up, probabilistic risk awareness is crucial. Therefore, it is important to be able to quarantine transcripts for which the statistics are driven by observations that do not fit the model assumptions. Those transcripts can be excluded from the study or can be given further attention with ad hoc analyses. For example, the statistics for a specific gene that include outliers for one or more biological replicates could be recalculated excluding those replicates from the analysis. In case the statistics such as *P*-value or fold change would dramatically differ (e.g. going from strongly significant to non-significant), the user should consider dropping such gene from further analyses. It is possible to identify outlier observations by analysing the distribution of residuals; however, in cases where limited biological replicates are available this analysis tends to be under-powered. The use of Bayesian inference allows a posterior predictive check, where the theoretical range of values for each observation is estimated by sharing the uncertainty across transcripts (e.g. the association of mean and overdispersion) and biological replicates (the sequencing depth unwanted variation).

Here, we propose a statistical framework for the detection of transcripts for which data do not fit the assumption of a negative binomial distribution, including deleterious outliers that bias the statistical inference toward false positives. This process includes two steps, where transcripts for which the statistics are biased by potential outliers are flagged and the likelihood of this event is calculated based on a truncated distribution, which helps control false positives. In principle, a one-step approach would also be possible, using a robust compound-Poisson distribution and generating the theoretical data distribution from a negative binomial distribution from the inferred mean and variance. We experimented with thicker tail distributions but could not find a numerically stable and computationally efficient distribution.

With ppcseq, the user can control for an arbitrary rate of false positives at the transcript level, which is a direct and intuitive measure of confidence. This method can be used to check and visualize results from all methods based on a negative binomial framework (e.g. edgeR and Deseq2) providing a more robust differentially abundant transcript set. ppcseq not only has broad applicability in bulk transcriptomic analysis but represents a foundation for future work with application to single-cell transcriptomics data and to other generalized linear models.

## DATA AVAILABILITY

The code used to conduct the analyses is available at github.com/stemangiola/ppcseq.

## Supplementary Material

lqab005_Supplemental_File
