# Correction to ‘Probabilistic outlier identification for RNA sequencing generalized linear models’

**DOI:** 10.1093/nargab/lqae024

**Published:** 2024-02-27

*NAR Genomics and Bioinformatics*, Volume 3, Issue 1, March 2021, lqab005, https://doi.org/10.1093/nargab/lqab005

In the originally published online version of this manuscript, there was an error in the bold heading for panel A in Figure 1.

The heading in Figure 1 should read:


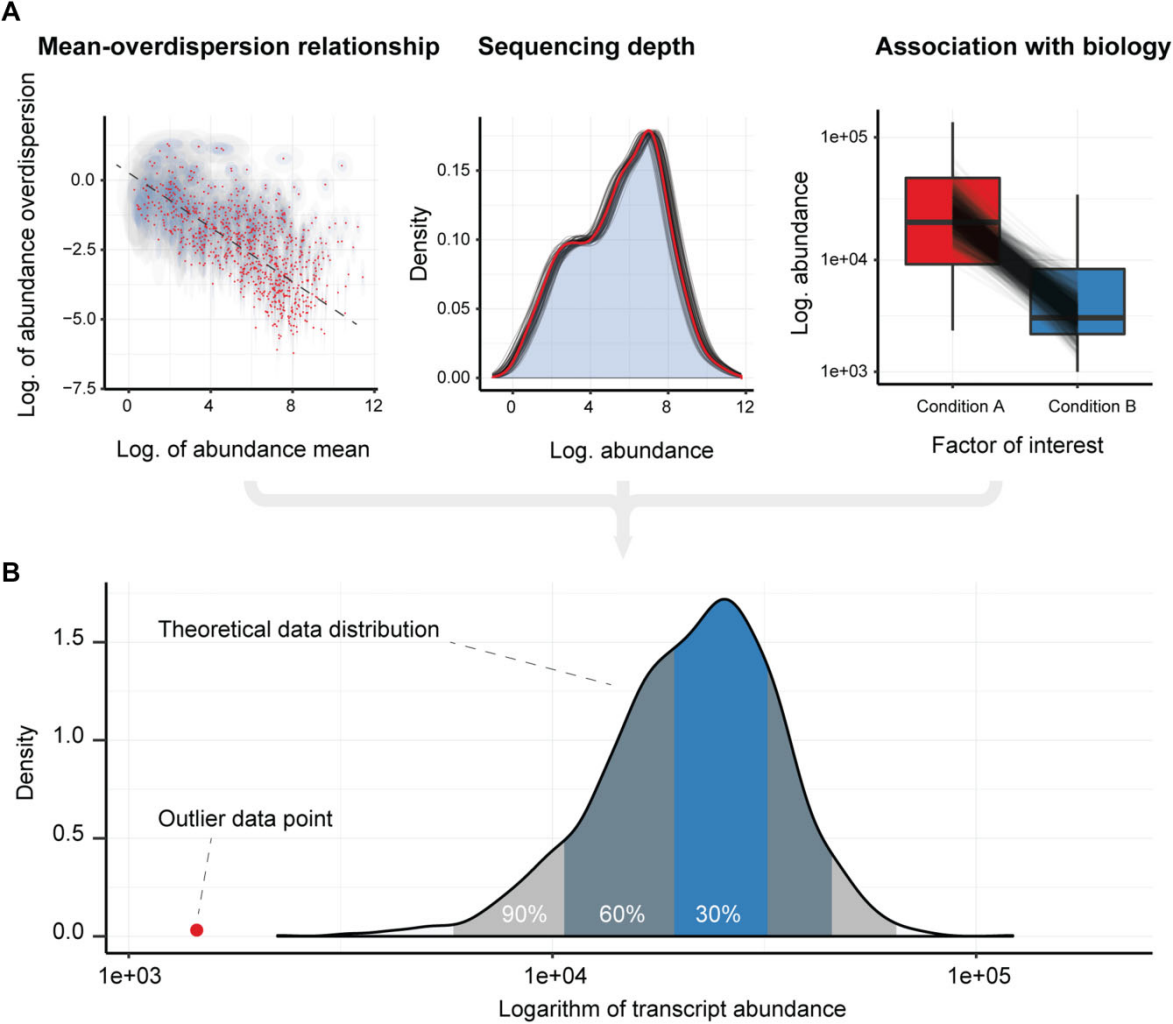


instead of:


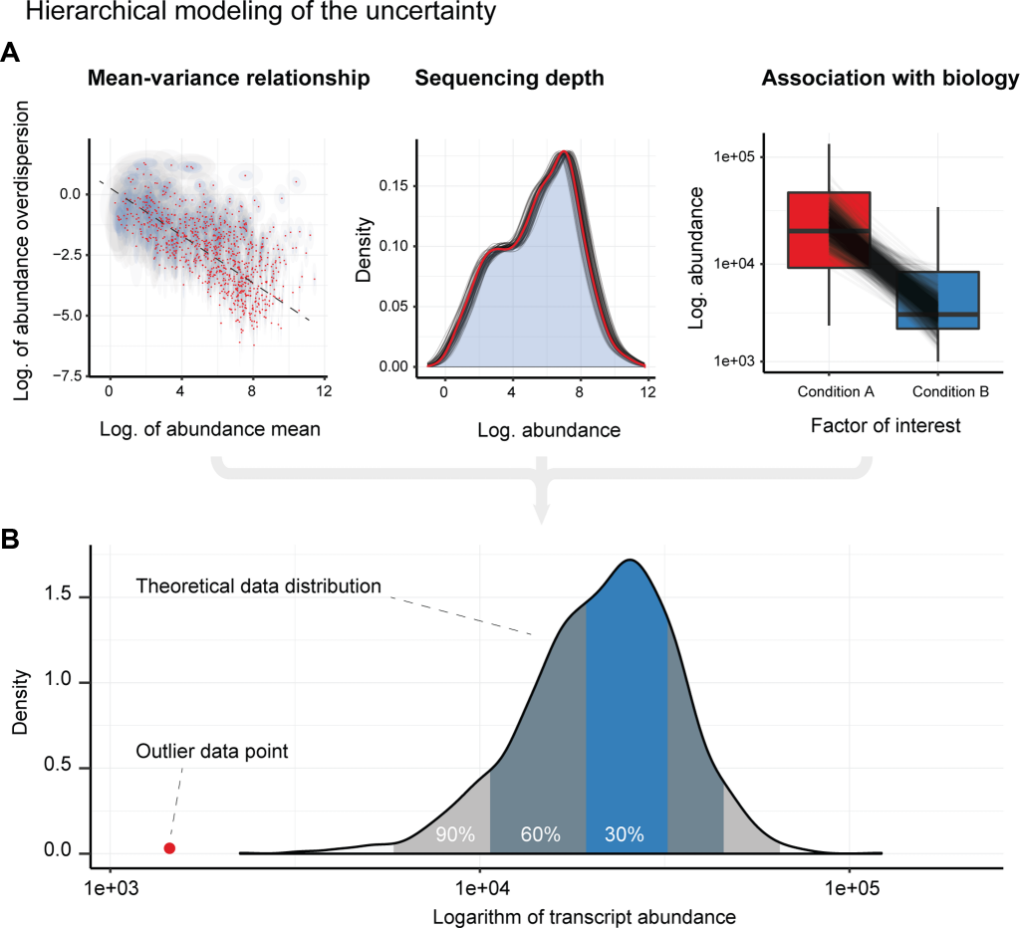


This error has been outlined only in this correction notice to preserve the version of record.

