# Supplementary material for: Probabilistic outlier identification for RNA sequencing generalized linear models
Source: NAR Genom Bioinform. 2021 Mar 1;3(1):lqab005. doi: 10.1093/nargab/lqab005 (PMC7936652; doi:10.1093/nargab/lqab005)
Supplement: lqab005_Supplemental_File [file lqab005_supplemental_file.docx]

# Supplementary methods

## Probabilistic framework

The probabilistic model can be represented by a likelihood function (Eq. 1) and a series of sampling statements (Eq. 2-7; Fig. S4), where the indices G, S, C represent genes, biological replicates (samples) and covariates respectively.


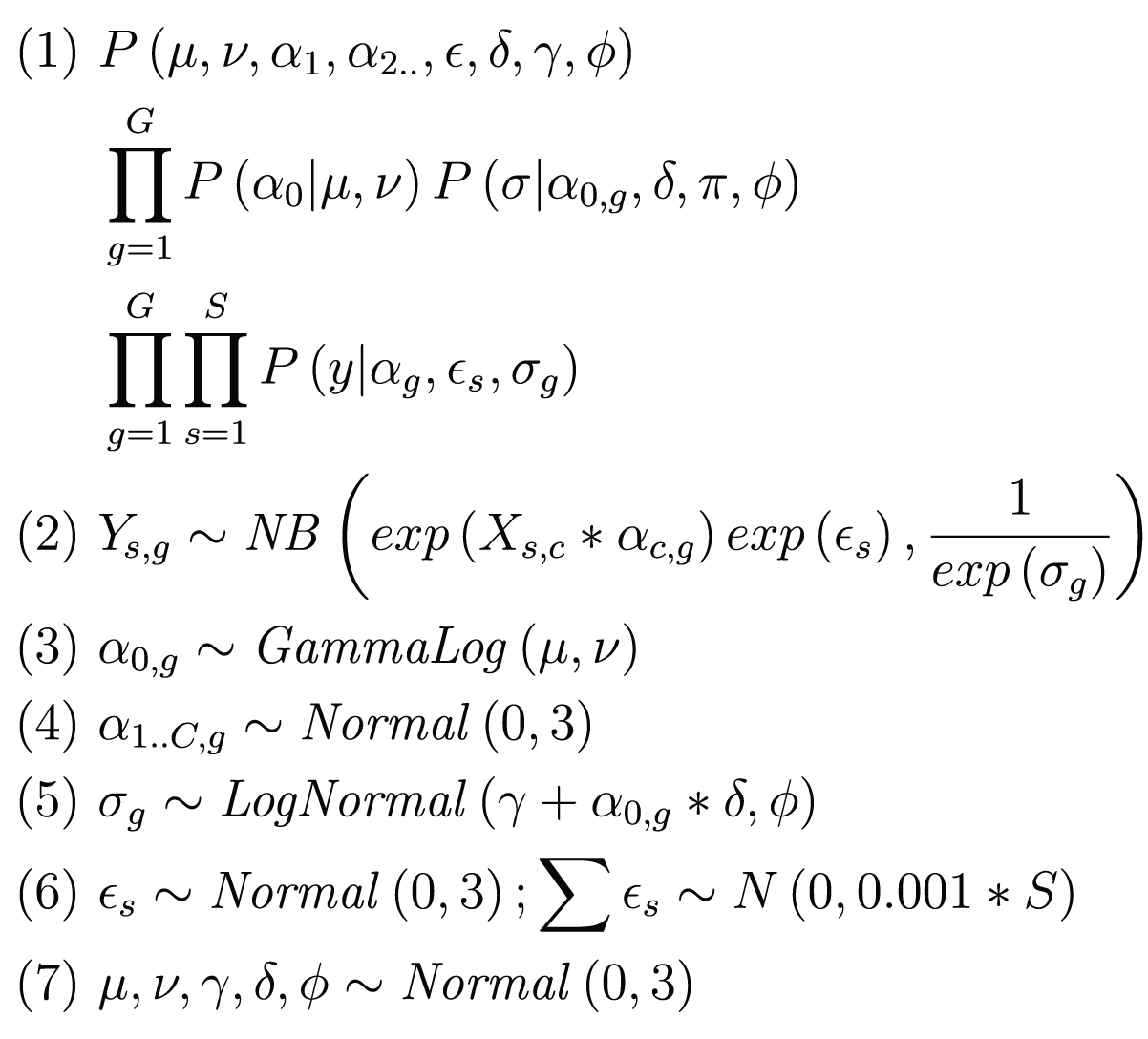


The negative binomial is parameterised by mean and overdispersion (Eq. 2). The mean is computed from the linear model where *X* represents the design matrix, α represents the covariate factors. The differences in sequencing depth across biological replicates are modelled with a biological replicate-wise exposure rate term ϵ that multiplies the transcripts expected abundance (mean). The parameter ϵ models a rate of sequencing depth. Being relative ϵ is given S-1 degrees of freedom (soft constraint to sum-to-zero according to Stan guidelines; mc-stan.org/docs). The exposure rates are inferred using a set of anchor genes selected from the bottom rank of differentially abundant gene transcripts provided by the user (n = 500 by default); the underlying assumption is that those genes are not associated with the factor of interest. This scaling principle is also used through diverse approaches (calculated before-hand) by some popular methods for differential gene-transcript abundance, such as DESEeq2 [(Love, Huber, and Anders 2014)](https://paperpile.com/c/q367pv/1I67) and edgeR [(McCarthy, Chen, and Smyth 2012; Robinson, McCarthy, and Smyth 2010)](https://paperpile.com/c/q367pv/lMTH+Nhh6). For example, the TMM method [(Robinson and Oshlack 2010)](https://paperpile.com/c/q367pv/szFi) used by edgeR calculates mean of the log expression ratios between each biological replicate and a reference, trimmed at the tails (at an arbitrary percentage) and weighted by the inverse of the approximate asymptotic variances. The algorithm DESeq2 uses scaling factors determined by the median ratio of gene counts relative to geometric mean per gene. The overdispersion is modelled gene-wise by the parameter σ. The inverse association between log transcript abundance and its negative log over-dispersion is modelled as a linear function with a negative slope (Eq. 5), where ɑ_0_ represents the log abundance (at the intercept of the linear equation; Eq. 2) and ɑ_1..C_ represent the independent regression coefficients; while 𝜸, 𝛿 and φ represent the intercept, slope and standard deviation of the negative log variance-log mean association. The expected abundance across genes is modelled as generated by a gamma distribution, where μ represents the mean of the gamma prior for expected transcript abundances, 𝛎 represents the variance of such prior. Equations 4, 6 and 7 represent non-informative hyperpriors, according to the parameters value ranges. Internally, the model is parameterised in logarithmic space for higher numerical stability. For the direct calculation of the credible intervals from the generated posterior distribution for the second inference step, the number of generated data drawn *D* from the model is chosen as sufficient to produce on average *N* draws (*N* = 10 by default) outside the credible interval of choice. The number of total draws needed is calculated as follows.

$$D =\frac{N}{p};$$

where $p = 0.05$, for the inference discovery step, $p= 2 *\frac{fpr}{biological replicates} ,$for the inference testing step, N is the desired number of draws outside the credible interval, and *fpr* is the desired false positive rate. For example, if we desire 50 draws outside the credible interval, a false positive rate of 0.1, and the dataset includes 10 biological replicates, we need to sample 2500 draws from the posterior distribution. From a theoretical perspective in case the data was strictly generated from a specific probabilistic model, these example values will provide 1% false outlier classifications. Given 100 genes to be tested, we can expect to obtain one false positive; while if we tested one gene, and repeated the analysis 100 times, we can expect to obtain one false positive overall. The multiplication by two for the testing step is to reflect that we are doing a one tail observation only (testing for deleterious outliers only).

For the second *test* step, the negative binomial distribution of transcript abundance is modelled as truncated at the quantiles 0.025 and 0.975 (corresponding to a 95% credible interval used to quarantine outlier data-points in the first step). Given the computationally demanding calculation of probability densities of a truncated negative binomial, we employ a heuristic adjustment to the over-dispersion of the generated quantities for the calculation of the predictive posterior credible intervals. That is, given that the under-estimation of over-dispersion that a standard negative binomial has compared to a truncated negative binomial at the 95% percentile is approximately constant for any combination of mean and over-dispersion ranges typical in RNA sequencing data (Fig. S1), we use the posterior distribution of mean and over-dispersion from a non-truncated negative binomial and then generate data adjusting the over-dispersion after fitting.

#

# Supplementary Tables and Figures

**Table S1.** Top rank differentially abundant transcripts (estimated with edgeR according to user guidelines) that included one or more outlier data points.

| Data set | Rank edgeR | Rank edgeR robust | Rank DESeq2 |
| --- | --- | --- | --- |
| GSE99374 CD8 | 1 | 7 | 1 |
| Mangiola et adipo | 2 | 27 | 1 |
| GSE141027 lipoma | 3 | 26 | 2 |
| GSE137631 muscle | 25 | 19 | 41 |
| Atkins et brain | 86 | 191 | 66 |
| GSE151005 arabidopsis | 429 | NA | NA |


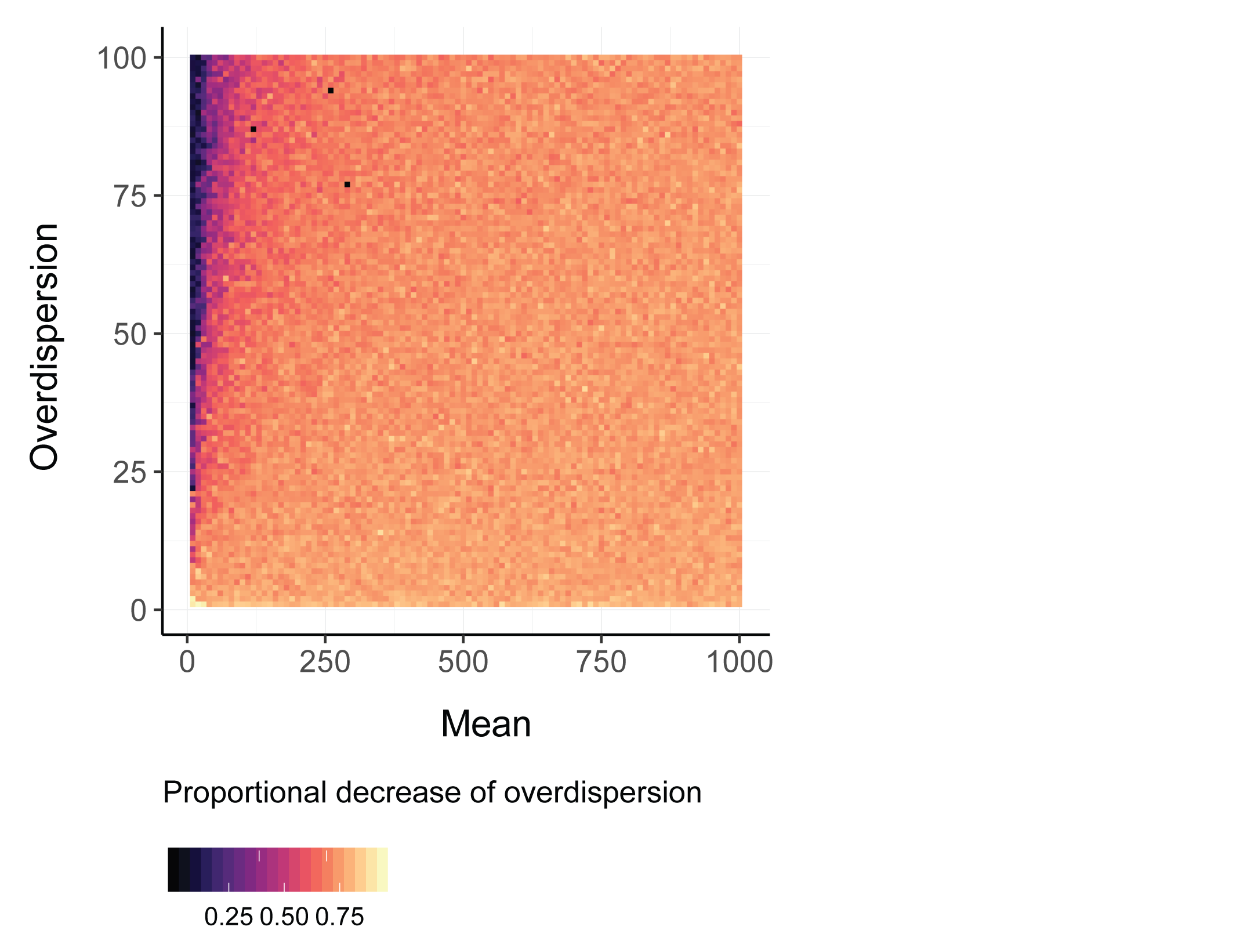


**Figure S1** Proportional decrease of over-dispersion of a negative binomial following a truncation of the distribution at the 2.5% and 97.5% percentiles. This figure shows that for a fixed truncation (e.g. using 95% percentile) the over-dispersion of a generic negative binomial distribution decreases of consistent amount, no matter what mean and over-dispersion parameters are (safely within the range of real-world data). This allowed us to compensate for truncation directly, avoiding the use of a truncated negative binomial distribution within the Bayesian inference model, as it is computationally very inefficient.


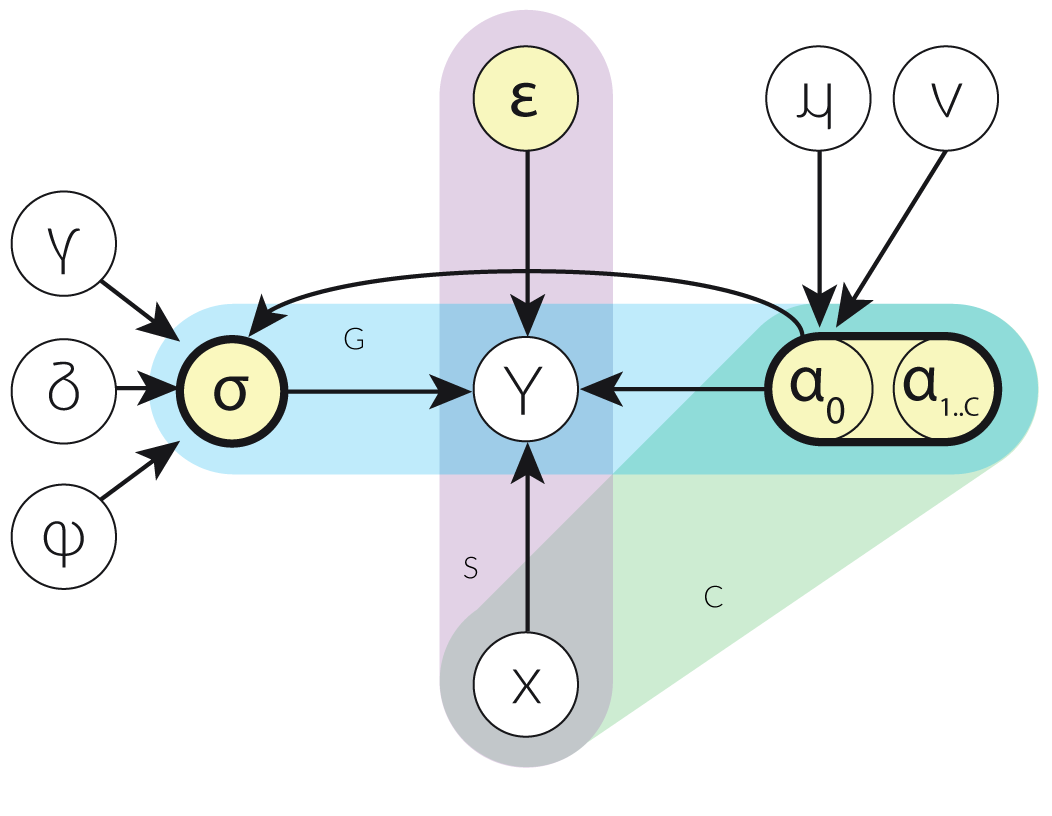


**Figure S2.** Graphical plated representation of the inference model representing Eq. 1-7. The white circles represent observed data. The yellow circles represent parameters (reals, vectors or matrices). The coloured frames group variables into a subgraph that repeats for transcripts/genes (G), biological replicates (S) and covariates (C).


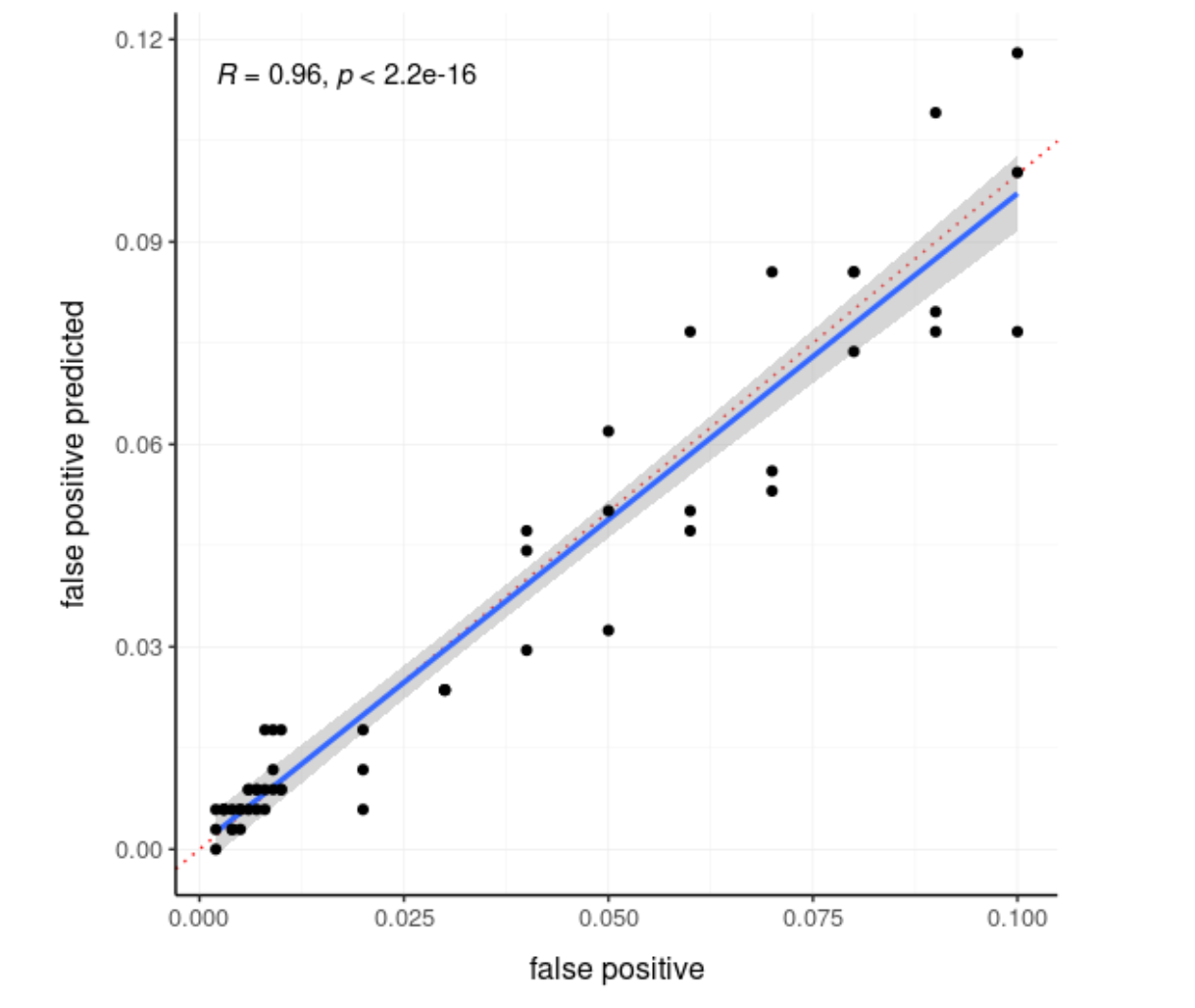


**Figure S3.** Calibration analysis for a linear model with one factor of interest and one further covariate (Formula: ~ Label + W). The false positive aimed significantly agrees with the ground-truth false positive. The source data (Mangiola_2018) and methods used for this experiment are described in the subsection “Calibration and accuracy test”. The code used to build this figure is included in the vignettes folder of the repository https://github.com/stemangiola/ppcseq.


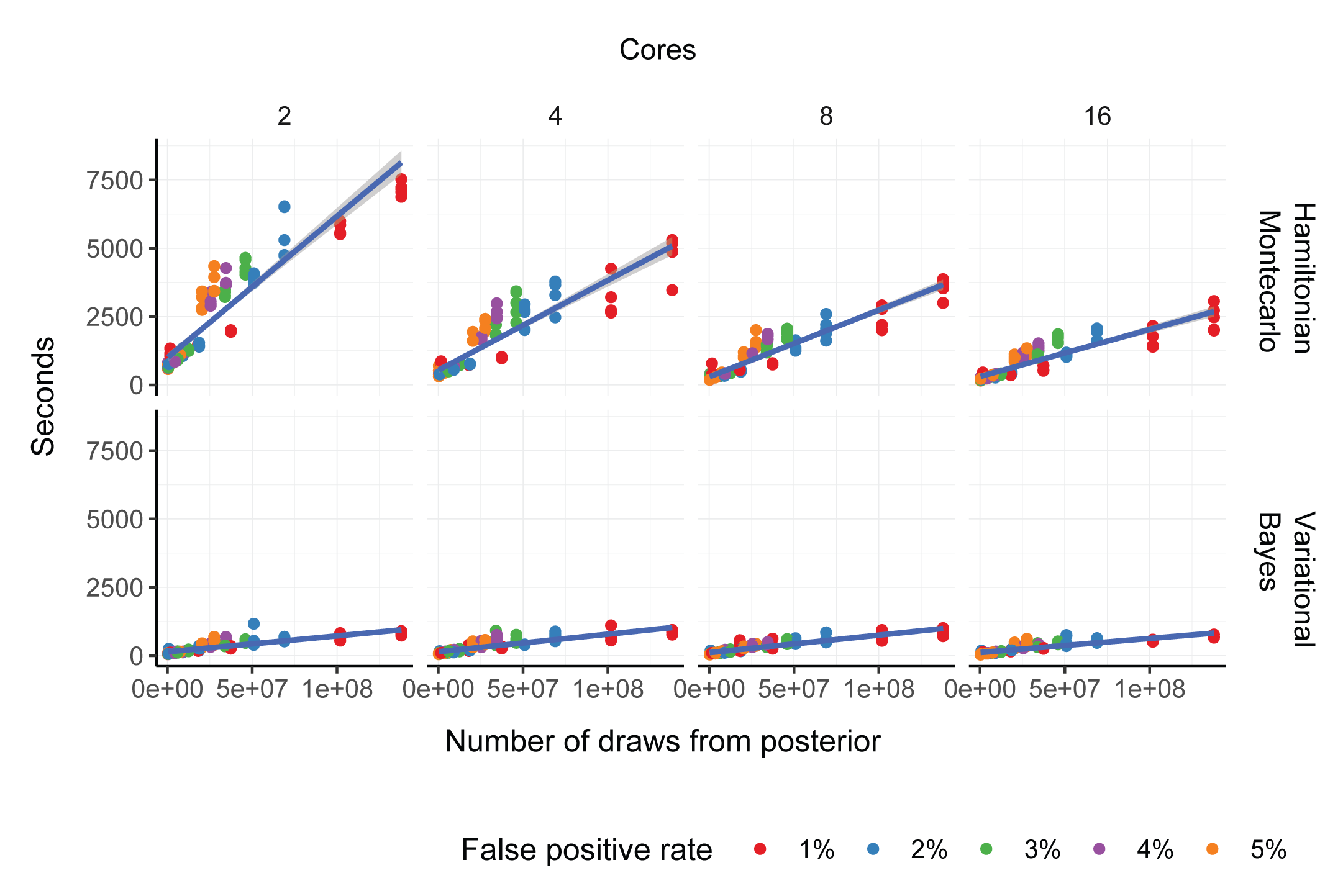


**Figure S4.** Benchmark of execution time across runs with diverse user-defined false positive rates, between full posterior sampling using Hamiltonian Monte Carlo and approximated posterior (multivariate normal) using variational Bayes.


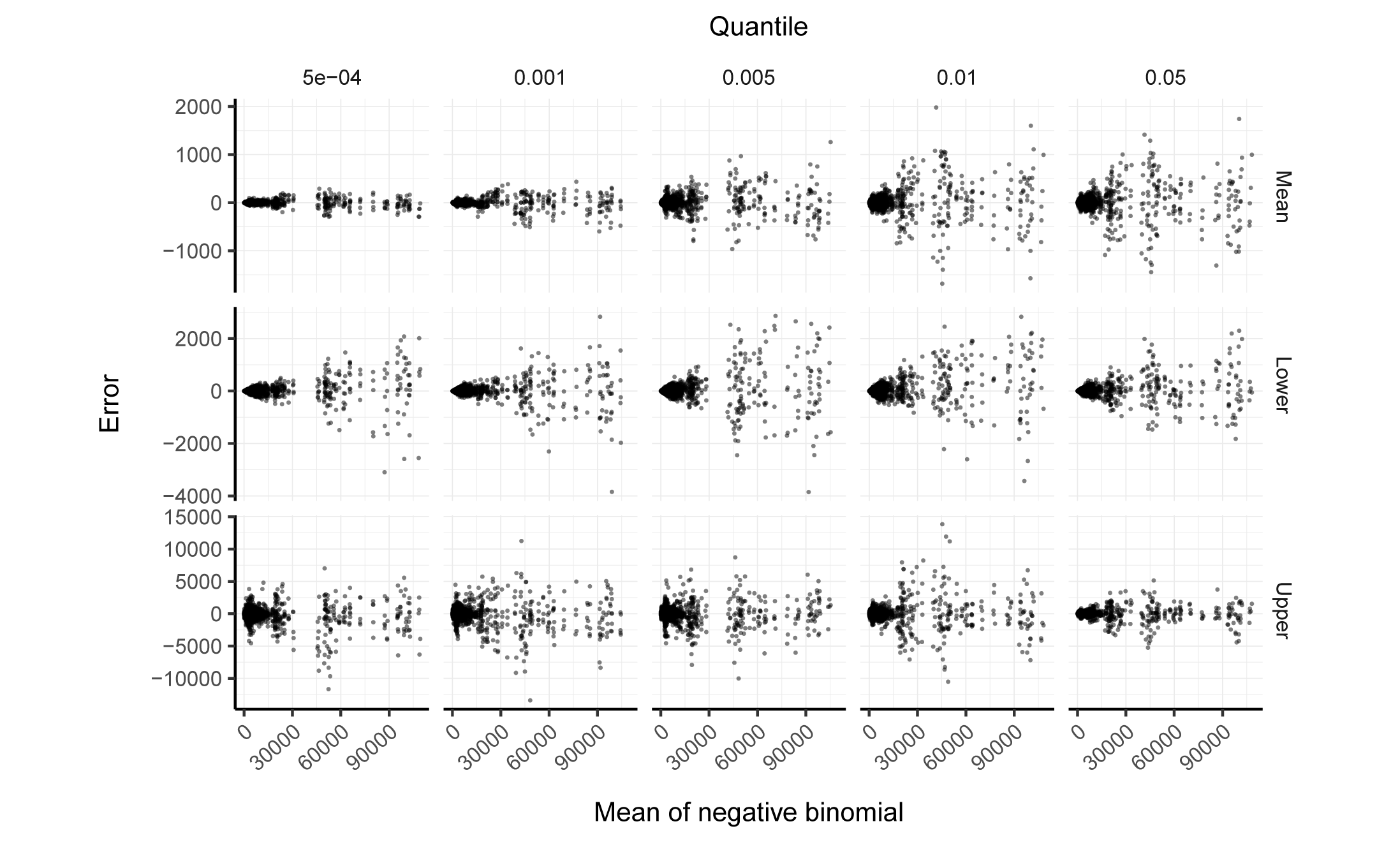


**Figure S5** Association between error between credible interval estimation using posterior draws (standard procedure) and using approximation (see Materials and Methods). This figure shows the absence of bias of credible interval estimation using the approximation method compared with the pure calculation based on posterior probability draws.

References

[Love, Michael I., Wolfgang Huber, and Simon Anders. 2014. “Moderated Estimation of Fold Change and Dispersion for RNA-Seq Data with DESeq2.” *Genome Biology* 15 (12): 550.](http://paperpile.com/b/q367pv/1I67)

[McCarthy, Davis J., Yunshun Chen, and Gordon K. Smyth. 2012. “Differential Expression Analysis of Multifactor RNA-Seq Experiments with Respect to Biological Variation.” *Nucleic Acids Research* 40 (10): 4288–97.](http://paperpile.com/b/q367pv/lMTH)

[Robinson, Mark D., Davis J. McCarthy, and Gordon K. Smyth. 2010. “edgeR: A Bioconductor Package for Differential Expression Analysis of Digital Gene Expression Data.” *Bioinformatics*  26 (1): 139–40.](http://paperpile.com/b/q367pv/Nhh6)

[Robinson, Mark D., and Alicia Oshlack. 2010. “A Scaling Normalization Method for Differential Expression Analysis of RNA-Seq Data.” *Genome Biology* 11 (3): R25.](http://paperpile.com/b/q367pv/szFi)
